# Ciprofloxacin Enhances TRAIL-Induced Apoptosis in Lung Cancer Cells by Upregulating the Expression and Protein Stability of Death Receptors through CHOP Expression

**DOI:** 10.3390/ijms19103187

**Published:** 2018-10-16

**Authors:** Eun Jin Lim, Yu Jeong Yoon, Jeonghoon Heo, Tae Hwa Lee, Young-Ho Kim

**Affiliations:** 1 Department of Molecular Biology and Immunology, College of Medicine, Kosin University, Busan 49267, Korea; eunjin1025@hanmail.net (E.J.L.); yuuun33@naver.com (Y.J.Y.); jeonghoonheo@kosin.ac.kr (J.H.); 2 Department of Obstetrics and Gynecology, College of Medicine, Kosin University, Busan 49267, Korea; leehula@kosin.ac.kr; 3 Institute for Medical Sciences, College of Medicine, Kosin University, Busan 49267, Korea

**Keywords:** ciprofloxacin, TRAIL, death receptor, CHOP

## Abstract

Ciprofloxacin (CIP) is a potent antimicrobial agent with multiple effects on host cells and tissues. Previous studies have highlighted their proapoptotic effect on human cancer cells. The current study showed that subtoxic doses of CIP effectively sensitized multiple cancer cells to tumor necrosis factor-related apoptosis-inducing ligand (TRAIL)-induced apoptosis. Although TRAIL alone mediated the partial proteolytic processing of procaspase-3 in lung cancer cells, co-treatment with CIP and TRAIL efficiently restored the complete activation of caspases. We found that treatment of lung cancer with CIP significantly upregulated the expression and protein stability of death receptor (DR) 5. These effects were mediated through the regulation of transcription factor CCAT enhancer-binding protein homologous protein (CHOP) since the silencing of these signaling molecules abrogated the effect of CIP. Taken together, these results indicated that the upregulation of death receptor expression and protein stability by CIP contributed to the restoration of TRAIL-sensitivity in lung cancer cells.

## 1. Introduction

Tumor necrosis factor (TNF)-related apoptosis-inducing ligand (TRAIL), a member of the TNF superfamily, is a potent apoptosis-inducing cytokine. TRAIL appears to specifically kill a wide variety of cancer cells in cultured and xenografted tumors but has little or no effect on normal cells [1,2,3]. TRAIL-induced apoptosis is associated with the interaction of TRAIL with two closely related membrane receptors, TRAIL-R1 and TRAIL-R2. This interaction results in the cooperation with the adaptor molecule Fas-associated protein with death domain (FADD), leading to the recruitment and cleavage of the initiator caspase-8 and the consequent activation of an effector caspase, such as caspase-3 [4,5]. However, many types of cancer develop TRAIL resistance, which is related to the high expression levels of decoy receptors and antiapoptotic proteins, mutations in TRAIL receptors, and dysregulation of death-inducing signaling complex (DISC) formation [6,7,8]. Cancer cells acquire TRAIL resistance through multiple unknown mechanisms. Therefore, it is important to identify therapeutic agents capable of sensitizing resistant cancer cells to TRAIL-induced apoptosis.

Ciprofloxacin (CIP) is a food and drug administration (FDA)-approved fluoroquinolone (FQ) widely used as a broad spectrum antibiotic. It is effective against various gram-positive and gram-negative bacteria, specifically by targeting bacterial DNA gyrase and topoisomerase [9,10]. Besides having antimicrobial activity, CIP reportedly exerts immunomodulatory effects in rodent models and human [11,12], improving a wide spectrum of conditions, including thrombocytopenia [13,14,15], Crohn’s disease [16,17], and rheumatoid arthritis [18,19]. As reported by previous studies, FQs, alone or in combination with other chemotherapeutic agents, have the ability to induce apoptosis and cell cycle arrest in various cancer cell lines, rendering them unique among other antibiotic family members [20,21,22,23,24]. In addition, although CIP was shown to stimulate TRAIL treatment in the lung cancer cell line A549, the molecular basis by which CIP sensitizes TRAIL-mediated apoptosis was not fully investigated yet.

The current study reported for the first time that CIP sensitized cancer cells to TRAIL-induced apoptosis and that the expression and stability of TRAIL receptor contributed to this sensitization. We showed that the CIP-induced expression and stability of TRAIL receptor was required to significantly enhance the sensitivity of cancer cells to TRAIL-induced apoptosis. Therefore, this study showed the importance of death receptors as targets for CIP to enhance the sensitivity of cancer cells to TRAIL-induced apoptosis.

## 2. Results

### 2.1. Ciprofloxacin Potentiated TRAIL-Induced Apoptosis in Human Lung Cancer Cells

**Figure 1:**
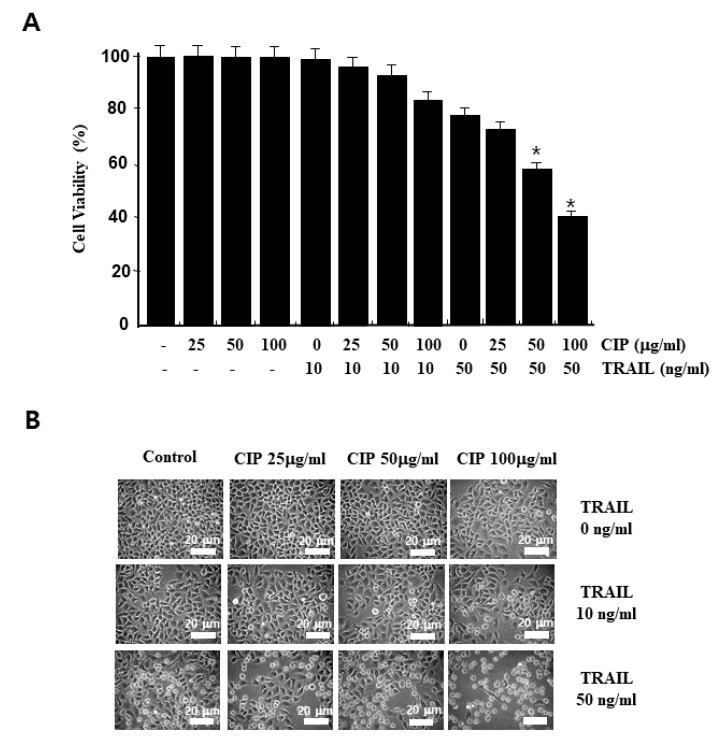

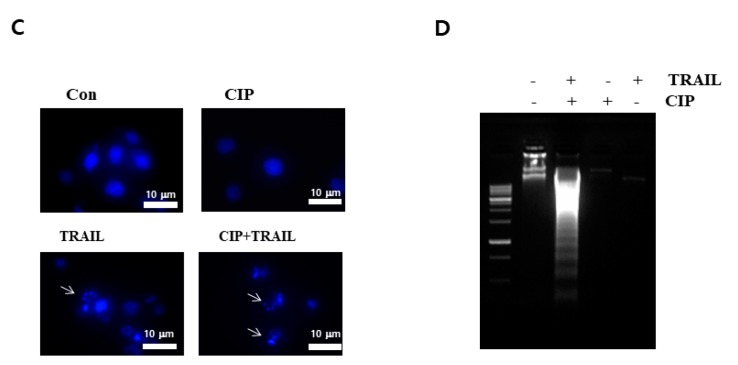
CIP enhanced TRAIL-induced A549 cell death. (**A**) Cells were pretreated with various concentrations of CIP for 20 h before exposure to TRAIL (10 and 50 ng/mL) for 4 h. Cell viability was analyzed by trypan blue exclusion assay. Data represent the mean ± SD of 3 samples. * *p* < 0.05 compared to the CIP + TRAIL-treated cells. (**B**) Cells were treated with TRAIL in the presence or absence of CIP for 24 h. After treatment, change in cell morphology was detected by light microscopy. Scale bar = 20 μm. (**C**) Microscopic examination was performed to detect apoptosis by nuclear staining with DAPI. The images shown are representatives of three independent experiments. Scale bar = 10 μm. (**D**) Cells were treated with TRAIL for 4 h in the presence or absence of CIP for 20 h. For analyzing DNA fragmentation, fragmented DNA was separated by using 1.5% agarose gel.

To investigate the effect of CIP on TRAIL-induced apoptosis, A549 cells were exposed to 10 to 50 ng/mL of TRAIL with or without CIP at various concentrations. Neither CIP nor TRAIL alone had any effect on cell death, but combined treatment with both CIP and TRAIL markedly enhanced cell death in a dose-dependent manner and also induced morphological changes in the cells (Figure 1A,B). Next, we examined whether combined treatment with CIP and TRAIL induces DNA fragmentation. In the cells that received the combined treatment with CIP and TRAIL, we detected the typical apoptotic nuclei (Figure 1C) and analyzed the DNA fragmentation (Figure 1D). These data indicated that combined treatment with CIP- and TRAIL-induced cell death in human lung cancer cells.

### 2.2. CIP Sensitized TRAIL-Induced Apoptosis through Caspase Pathway

**Figure 2:**
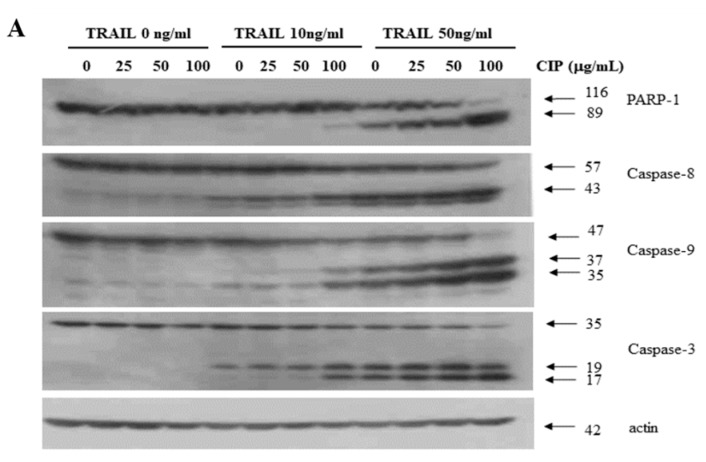

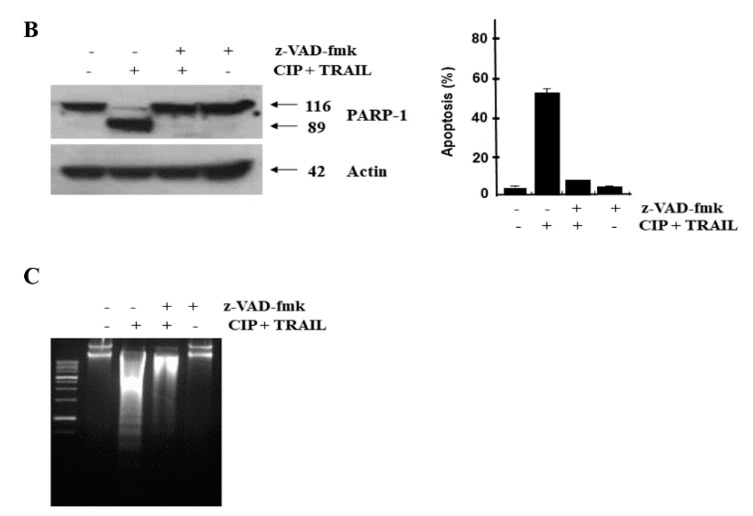
CIP treatment-induced caspase activation in A549 cells. (**A**) The protein expression of caspase-3, caspase-8, caspase-9, caspase-7, and PARP after treatment with different doses of CIP+TRAIL for 24 h. The total cells were collected and the lysates were subjected to western blotting with specific antibodies. Actin was used as a loading control. The proteolytic cleavages in PARP, cas-3, cas-8, cas-7, and cas-9 are indicated by arrows. (**B**) A549 cells were incubated with 50 µM z-VAD-fmk for 1 h before treatment with CIP + TRAIL. Equal amounts of cell lysates (40 µg) were electrophoresed and analyzed for PARP-1 by western blotting. The proteolytic cleavage of PARP is indicated by an arrow. (**C**) For analyzing DNA fragmentation, fragmented DNA was separated by using 1.5% agarose gel.

To evaluate the mechanism of CIP and TRAIL-induced apoptosis activation, poly (ADP-ribose) polymerase (PARP) cleavage and caspase activity were determined in the presence of TRAIL, CIP, or both. Figure 2A shows that in the presence of TRAIL, PARP was cleaved, yielding a characteristic 85 kDa fragment. The combination treatment of TRAIL and CIP also resulted in elevated activation of caspase-8, caspase-9, and caspase-3. In addition, we showed that TRAIL- and CIP-induced apoptosis was blocked by Benzyl carbonyl-Val-Ala-Asp-fluoromethyl ketone (z-VAD-fmk) peptide, a general caspase inhibitor (Figure 2B). We also found that z-VAD-fmk prevented the increase in apoptotic DNA accumulation due to treatment with CIP and TRAIL (Figure 2C). These results provided further evidence that TRAIL induced the sensitization of cancer cells to CIP through a caspase-dependent pathway.

### 2.3. CIP Upregulated Death Receptors Expression in Various Cancer Cells

**Figure 3:**
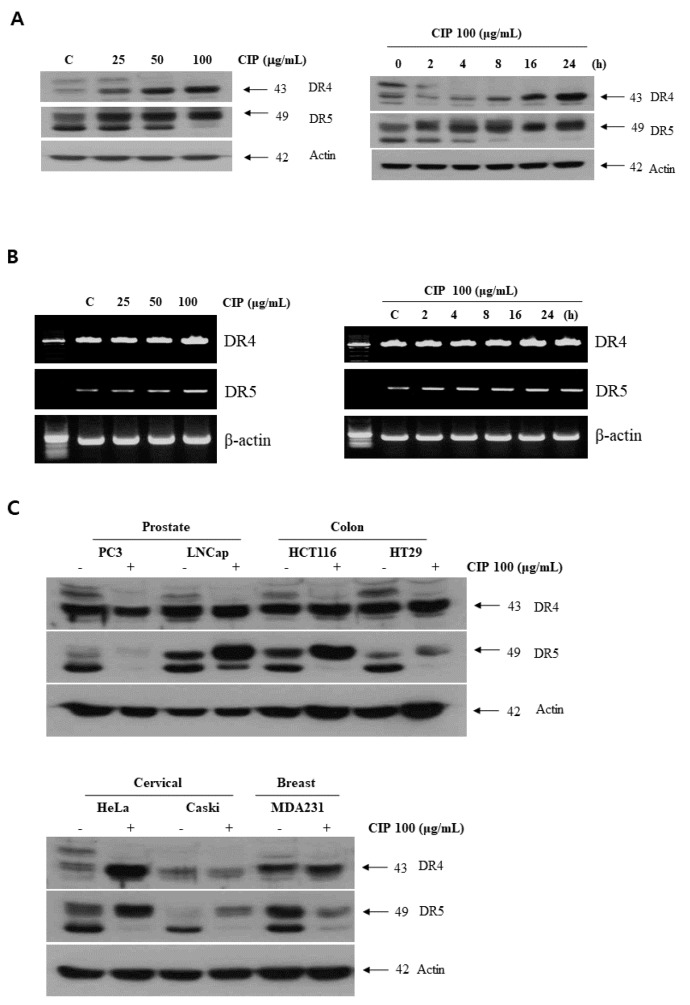
CIP-induced DR5 and DR4 expression. (**A**) A549 cells were treated with various concentrations of CIP (**left**) and with CIP 100 µg/mL for various time periods (**right**). Whole cell extracts were analyzed for DR4 and DR5 expression by western blotting. (**B**) CIP-induced DR4 and DR5 gene expression. A549 cells were treated with various concentrations of CIP (**left**) and with CIP 100 µg/mL for various time periods (**right**). Total RNA was extracted and examined for DR4 and DR5 expression by RT-PCR. Actin was used as an internal control to show equal RNA loading. (**C**) Various cancer cell lines were treated with CIP for 24 h and whole cell extracts were analyzed by western blotting. Equal amounts of protein (40 µg) were separated by SDS-PAGE and immunoblotted.

We determined whether the modulation of DR4 and/or DR5 protein levels was involved in the sensitizing effect of CIP on TRAIL-induced apoptosis in lung cancer cells. Figure 3 shows that CIP-regulated, TRAIL-induced apoptosis corresponded with upregulation of DR4 and DR5. DR4 and DR5 expression levels in lung cancer cells were increased in a time- and dose-dependent manner by CIP treatment (Figure 3A). Reverse transcription (RT)-PCR analysis showed that CIP treatment slightly increased DR5 mRNA levels in a dose- and time-dependent manner, but not those of DR4 (Figure 3B). We also investigated whether the CIP-induced upregulation of DR5 and DR4 is specific to A549 cells or also occurs in other lung cancer cell types (Figure S2). Prostate cancer cells (PC3 and LNCaP), colon cancer cells (HCT116 and HT29), cervical cancer cells (HeLa and Caski), and breast cancer cells (MDA231) were exposed to CIP (100 μg/mL) for 24 h and then examined for DR5 and DR4 protein expression. CIP induced the expression of DR5 (Figure 3C, middle panel) in the LNCaP, HCT116, HeLa, and Caski cells. No significant induction of DR5 expression occurred in the PC3, HT29, and MDA 231 cells. These findings suggested that the CIP-induced upregulation of DR5 and DR4 is not cell type-specific.

### 2.4. Death Receptor Was Required by CIP to Enhance TRAIL-Induced Apoptosis

**Figure 4:**
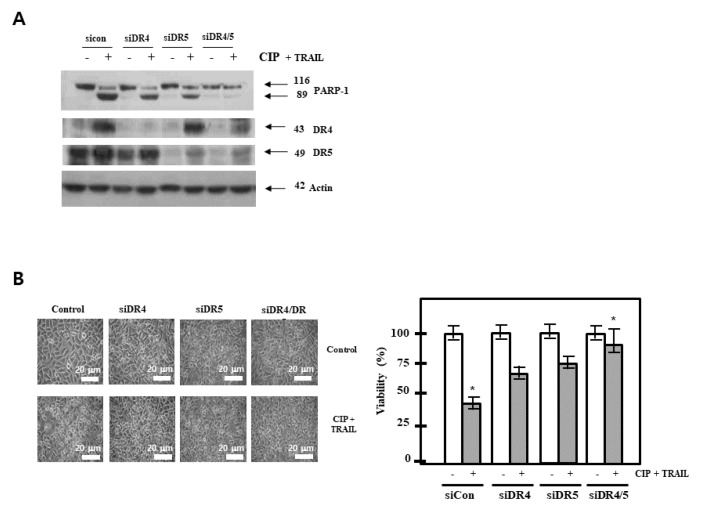
Effect of death receptors knockdown on CIP-induced sensitization to TRAIL. (**A**) A549 cells were transfected with DR5 siRNA, DR4 siRNA, and combined DR5 and DR4 siRNA. After 48 h, the cells were pretreated with CIP for 20 h and then treated with TRAIL for 4 h. Whole cell extracts were analyzed by western blotting using antibodies against PARP, DR4, and DR5. (**B**) Cell death was determined by Annexin V/PI staining. Scale bar = 20 μm. The bar represents the mean ± SD. * *p* < 0.01 indicates a significant difference between the untreated control and CIP + TRAIL-treated samples.

To determine the role of DR5 and DR4 in TRAIL-induced apoptosis, we used siRNAs specific to DR5 and DR4 to downregulate their expression. The siRNAs for DR4 and DR5 reduced CIP-induced DR4 and DR5 expression, respectively, more than that by the control siRNA (Figure 4A). However, DR4 siRNA and DR5 siRNA had minimal effects on the CIP-induced upregulation of DR4 and DR5. Next, by using immunoblotting (Figure 4A) and Annexin V staining assay (Figure 4B), we examined whether the suppression of DR4 and/or DR4 by siRNA can abrogate the sensitizing effects of CIP on cancer cells to TRAIL-induced apoptosis. The results revealed that the effect of CIP on TRAIL-induced apoptosis was effectively abolished in cells transfected with both DR5 and DR4 siRNAs, whereas treatment with only DR4 siRNA or only DR5 siRNA had no effect on TRAIL-induced apoptosis. The silencing of both receptors abolished apoptosis to the same degree as the silencing of either DR4 or DR5, suggesting that both DR4 and DR5 played major roles in TRAIL-induced apoptosis.

### 2.5. CHOP Mediated CIP-Induced Death Receptors Upregulation and Sensitization to TRAIL

**Figure 5:**
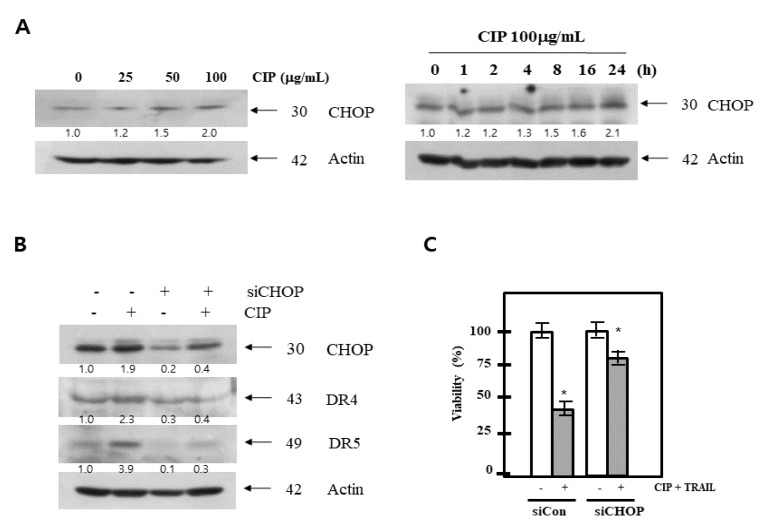
CIP exposure led to the regulation of CHOP proteins expression. (**A**) The effects of CIP on the expression levels of CHOP proteins. A549 cells were treated with various concentrations of CIP. Equal amounts (40 µg) of cell lysates were separated by SDS-PAGE. The images shown are representatives of two additional experiments which yielded similar results. (**B**) A549 cells were transfected with CHOP siRNA. After 48 h, the cells were pretreated with CIP for 24 h. Whole cell extracts were analyzed by western blotting using antibodies against CHOP, DR4, and DR5. (**C**) A549 cells were transfected with CHOP siRNA. After 48 h, cells were treated with CIP + TRAIL for 24 h. Cell death was determined by Annexin V/PI staining. The bar represents the mean ± SD. * *p* < 0.01 indicates a significant difference between the untreated group and CIP + TRAIL-treated samples.

Because CCAAT/enhancer-binding protein homologous protein (CHOP) is known to be a prominent endoplasmic reticulum (ER) stress marker and an important transcription factor of DR5, we next determined whether CIP induces CHOP expression. To further investigate the underlying mechanism of DR5 expression by CIP-induced CHOP upregulation, the protein levels of DR5 were also determined using CHOP siRNA. Our results showed that CHOP protein levels were significantly increased in a dose- and time-dependent manner (Figure 5A). In a parallel experiment, we observed similar results when other tumor cells were similarly treated with CIP (data not shown). In order to determine the effect of CHOP on CIP-induced apoptosis, we demonstrated the effects of CIP on CHOP siRNA-induced DR5 and DR4 expression. To clarify the functional role of CHOP in CIP-induced DR5 and DR4 upregulation, CHOP siRNA was also tested. DR5 was upregulated by CIP in A549 cells transfected with scrambled negative control RNA, but transfection with CHOP siRNA significantly abrogated the upregulation of DR5 (Figure 5B). In addition, treatment with CIP did not affect actin, which was used as a housekeeper gene in these experiments. As expected, CIP/TRAIL-induced cell death was substantially blocked by siCHOP (Figure 5C). These findings indicated that CIP-enhanced DR5 expression and TRAIL-induced apoptosis through CHOP activation.

### 2.6. CIP Sustained Death Receptor Protein Stability

**Figure 6:**
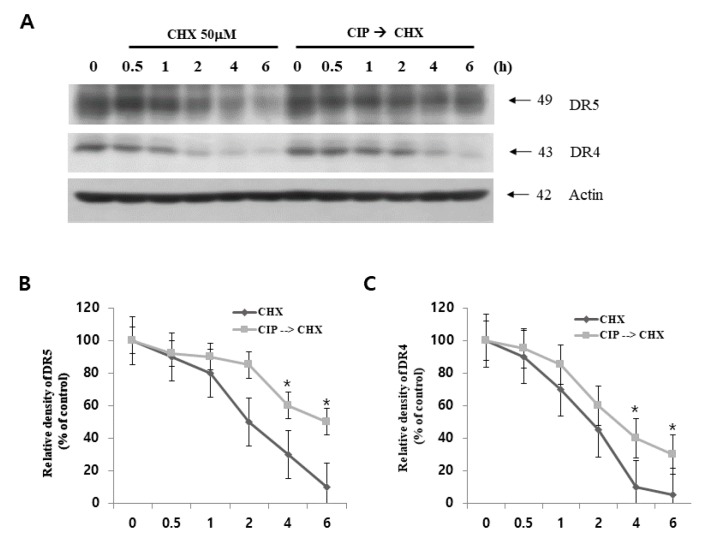
Effect of CIP on DR4 and DR5 protein stability. (**A**) A549 cells were treated with or without CIP for 24 h in the presence or absence of CHX for the indicated time periods. DR5, DR4, and actin protein levels were determined by western blotting. Actin was used as a loading control. The band intensity of DR5 (**B**) and DR4 (**C**) proteins was measured using the public domain JAVA image-processing program Image J. Each value represents the mean ± SD of three independent experiments. * *p* < 0.05 compared to that of CHX.

To characterize the mechanism underlying CIP-induced DR4/DR5 upregulation, we examined the effect of CIP on DR4/DR5 protein stability in A549 cells. After the treatment with CIP for 12 h, the cells were treated with cycloheximide (CHX), an inhibitor of *de novo* protein synthesis in the presence or absence of CIP. CHX gradually decreased DR4 and DR5 protein expression, but co-treatment with CHX and CIP sustained DR4 and DR5 protein expression (Figure 6). When DR5 is degraded via the ubiquitin-proteasome pathways, ubiquitination of DR5 by Cbl E3 ligase is important [25]. We found that the protein expression of Cbl, one of the E3 ligases that target DR5, was not markedly decreased in a time-dependent manner in CIP-treated cells (data not shown). These findings suggested that CIP induced the upregulation of death receptors expression at the post-translational level.

## 3. Discussion

This study showed for the first time that CIP enhanced TRAIL-mediated apoptosis in cancer cells. We revealed an effect of CIP on apoptosis signaling in the A549 lung cancer cells. We also found that CIP and TRAIL, at physiologically relevant doses currently being used for the treatment of antibacterial infections in human [26], caused cell cytotoxicity and apoptosis in A549 cells in a dose- and time-dependent manner.

CIP is an FDA-approved fluoroquinolone widely used as an antimicrobial. CIP has various anticancer activities, including growth-inhibitory and apoptosis-inducing activities. CIP inhibits cell growth mainly in colon, leukemia, prostate, and bladder cancers [21,27,28], and induces G2/M arrest mainly in bladder and prostate cancer cells [26,29]. CIP also induces apoptosis through the activation of p21 in bladder cells [26]. We showed by flow cytometry that CIP-induced G2/M arrest in lung cancer cells (data not shown). However, CIP is used as a sensitizer or anticancer drug-enhancer against chemotherapy-resistant cancer cells.

Although TRAIL is one of the potent cytokines with the potential to kill cancer cells selectively, its use has limitations because of the resistance that certain cancer types develop [30]. Therefore, safe agents that can sensitize cancer cells to TRAIL are needed. In this study, we provided evidence that CIP, an antimicrobial agent, sensitized human lung cancer cells to TRAIL. The overexpression of DR5 in TRAIL-resistant cancer cells restored TRAIL sensitivity [31]. Our results showed that CIP caused a significant increase in DR5 protein levels in lung cancer cells. Interestingly, CIP treatment did not affect the levels of other IAP (XIAP, cIAP1, and cIAP2) (data not shown), emphasizing the specific effect of CIP on DR5 expression. The increased level of DR5 expression, which led to the stimulation of the death receptor pathway, appeared to be the cause of the activation of caspase-3, caspase-8, caspase-9, and PARP (Figure 2). However, the enhancement of TRAIL-induced apoptosis by CIP-induced DR5 upregulation is associated with the increased activation of the caspase pathway [32].

We found that one of the most important upstream signals required for the upregulation of death receptors was reactive oxygen species (ROS). We found that CIP did not affect the generation of ROS in lung cancer cells that ROS scavengers did not downregulate the CIP-induced expression of DR5, and that ROS scavengers also did not abrogate the potentiation of TRAIL-induced apoptosis by CIP (data not shown). CHOP is thought to be one of the most important transcription factors that can directly bind to the DR5 promoter region and dynamically regulate DR5 expression [33]. We investigated whether the transcription factor CHOP is involved in the increase of DR5 expression by CIP. CIP increased translocation into the nucleus of CHOP. (Figure S1) We further revealed that the induction of DR5 by CIP is mediated through CHOP upregulation. These findings are supported by previous studies reporting the CHOP-upregulating and TRAIL effects-enhancing abilities of CIP [34].

Taken together, our study showed that CHOP might play a crucial role in the CIP-induced DR5 upregulation and cancer cell death. Hence, CIP could be an attractive candidate as a therapeutic agent for cancer chemotherapy. Future clinical studies in relevant animal models, however, are needed to completely elucidate the potential use of this fascinating molecule in the prevention and treatment of cancer.

## 4. Materials and Methods

### 4.1. Materials

RPMI 1640 medium, fetal bovine serum (FBS), penicillin, streptomycin, and all other tissue culture reagents were obtained from GIBCO/BRL Life Technologies (Grand Island, NY, USA). CIP was acquired from Aldrich (Milwaukee, WI, USA). Antibodies against PARP-1, Bcl-2, Bcl-xL, Mcl-1, and actin were purchased from Santa Cruz Biotechnology (Santa Cruz, CA, USA). Anti-phospho-JNK, anti-phospho-p38, and anti-phospho-ERK were purchased from Cell Signaling (Beverly, MA, USA). Benzyl carbonyl-Val-Ala-Asp-fluoromethyl ketone (z-VAD-fmk) was purchased from Biomol (Plymouth Meeting, PA, USA), 2’,7’-dichlorodihydrofluorescein diacetate (H_2_DCFDA) was purchased from Molecular Probes (Eugene, OR, USA). *N*-acetylcysteine (NAC) and all other chemicals used in this study were purchased from Sigma-Aldrich (St. Louis, MO, USA).

### 4.2. Cell Culture and Chemical Treatments

Human lung cancer cell line A549 was obtained from the American Type Culture Collection (Rockville, MD, USA). The A549 cells were cultured in RPMI 1640 medium supplemented with heat-inactivated 10% fetal bovine serum (FBS), penicillin (100 units/mL), and streptomycin (100 units/mL) at 37 °C in a humidified incubator with 5% CO_2_ and 95% air. When the cells were subconfluent, the medium was replaced with fresh medium, 25–100 ng/mL of CIP was added to the culture medium, and the cells were incubated for 24 h.

### 4.3. Cellular Viability Assay

For the morphological evaluation of cell death, approximately 5 × 10^5^ A549 cells were plated into each well of 60-mm cell culture dishes overnight. For the trypan blue exclusion assay, trypsinized cells were pelleted and resuspended in 0.2 mL of medium, 0.5 mL of 0.4% trypan blue solution, and 0.3 mL of phosphate-buffered saline solution (PBS). The samples were mixed thoroughly, incubated at room temperature for 15 min, and examined under a light microscope. At least 300 cells were counted for each survival determination.

### 4.4. Flow Cytometric Assay of DNA Content

After the treatment of cells with the indicated agent, the cells were harvested via trypsinization, fixed with 70% (*v*/*v*) alcohol at 4 °C for 30 min, and washed with PBS. After centrifugation, the cells were incubated in 0.1 mL of phosphate-citric acid buffer (0.2 M NaHPO_4_, 0.1 M citric acid, pH 7.8) for 30 min at room temperature. Next, the cells were centrifuged and resuspended with 0.5 mL of 50 µg/mL PI diluted in phosphate-citric acid buffer. DNA content was analyzed by using a FACScan flow cytometer (Beckman Coulter, Inc., Hialeah, FL, USA)

### 4.5. Analysis of Cytochrome C Release

A total of 2 × 10^6^ cells was harvested, washed once with ice-cold PBS, and gently lysed for 2 min in 100 µL ice-cold lysis buffer. Lysates were centrifuged at 13,000× *g* at 4 °C for 20 min to obtain the supernatants (mitochondria-free cytosolic extracts) and the pellets (mitochondria-containing fractions). The resulting cytosolic fractions were used for western blot analysis with an anticytochrome c antibody.

### 4.6. Western Blot Analysis

For western blot analysis, A549 cells were lysed with 1× Laemmli lysis buffer (2.4 M glycerol, 0.14 M Tris, pH 6.8, 0.21 M SDS, 0.3 mM bromophenol blue) and boiled for 10 min. Protein content was measured with the BCA Protein Assay Reagent (Pierce, Rockford, IL, USA). The samples were diluted with 1× Laemmli lysis buffer containing 1.28 M β-mercaptoethanol and equal amounts of protein were loaded on 8–12% SDS-polyacrylamide gels. Proteins were separated by SDS-PAGE and electrophoretically transferred to a nitrocellulose membrane. The nitrocellulose membrane was blocked with 5% nonfat dry milk in PBS-Tween 20 (0.1%, *v*/*v*) for 1 h. The membrane was incubated with a primary antibody (diluted according to the manufacturer’s instructions) at room temperature for 1.5 h. Horseradish peroxidase-conjugated antirabbit or antimouse IgG was used as secondary antibodies. Immunoreactive protein was visualized by the chemiluminescence protocol (ECL, Amersham, Arlington Heights, IL, USA). To ensure equal protein loading, each nitrocellulose membrane was stripped and reprobed with an antiactin antibody after the experiment was complete.

### 4.7. DNA Fragmentation and 4’,6’-Diamidino-2-Phenylindole (DAPI) Staining Assay

To assess DNA fragmentation after CIP with TRAIL for 24 h, approximately 1 × 10^6^ treated A549 cells were lysed for 30 min on ice in buffer containing 10 mM Tris (pH 7.4), 150 mM NaCl, 5 mM EDTA, and 0.5% Triton X-100. Lysates were vortexed and cleared by centrifugation at 13,000× *g* for 30 min. Fragmented DNA in the supernatant was extracted with an equal volume of a mixture of neutral phenol:chloroform:isoamyl alcohol (25:24:1) and electrophoresed on 1.5% agarose gels containing 0.1 g/mL EtBr. The cells were fixed on slide glass through the application of 4% paraformaldehyde for 30 min at room temperature. After washing with PBS, 300 nM DAPI was added to the fixed cells for 10 min. The cells were examined by fluorescence microscopy. Apoptotic cells were identified by the condensation and fragmentation of their nuclei. All microscopic examination of the DAPI-stained cells were performed in duplicate.

### 4.8. Data Analysis

All experiments were repeated three or more times. The data are represented as the mean ± standard deviation (SD). The difference between two mean values was analyzed using Student’s *t*-test and was considered statistically significant when *p* < 0.05.

## Supplementary Materials

Supplementary materials can be found at http://www.mdpi.com/1422-0067/19/10/3187/s1.

## Abbreviations

TRAIL	Tumor necrosis factor (TNF)-related apoptosis-inducing ligand
FADD	Fas-associated protein with death domain
DISC	Death-inducing signaling complex
FQ	Fluoroquinolone
CHOP	CCAAT/enhancer-binding protein homologous protein
CHX	Cycloheximide
PARP	Poly (ADP-ribose) polymerase
PI	Propidium iodide
NAC	*N*-acetyl-l-cyteine
ROS	Reactive oxygen species
