# Supplementary material for: Ciprofloxacin Enhances TRAIL-Induced Apoptosis in Lung Cancer Cells by Upregulating the Expression and Protein Stability of Death Receptors through CHOP Expression
Source: Int J Mol Sci. 2018 Oct 16;19(10):3187. doi: 10.3390/ijms19103187 (PMC6214089; doi:10.3390/ijms19103187)
Supplement: Supplementary file 1 [file ijms-19-03187-s001.pdf]

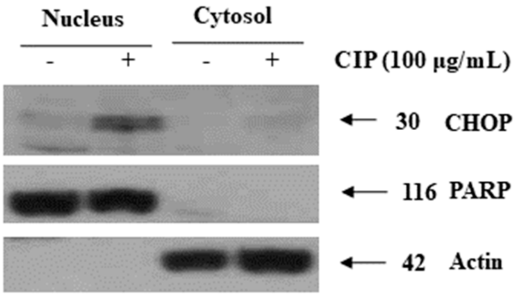

Figure S1. CIP exposure translocation of CHOP protein in nucleus. A549 cells were cultured in the presence or absence of 100 µg/ml CIP for 24 h and fractionated. The filters were also probed for PARP and Actin to show successful fractionation to nuclear and cytoplasmic fractions, respectively.

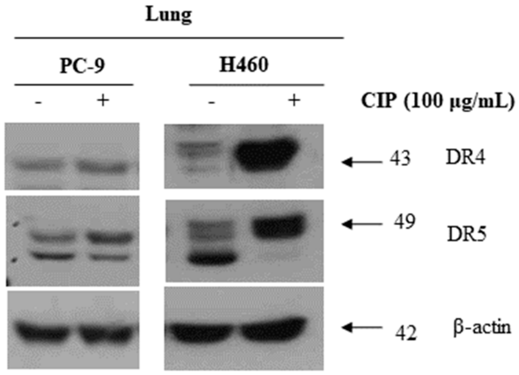

Figure S2. CIP-induced DR5 and DR4 expression in lung cancer cells. PC-9 and H460 cancer cells were treated with CIP for 24 h and whole cell extracts were analyzed by western blotting. Equal amounts of protein (40 µg) were separated by SDA-PAGE and immunoblotted.
